# Development and validation of a clinical score to identify hospitalised patients at high risk of drug-related problems

**DOI:** 10.1080/20523211.2025.2557876

**Published:** 2025-09-23

**Authors:** Kulchalee Deawjaroen, Jutatip Sillabutra, Nalinee Poolsup, Derek Stewart, Naeti Suksomboon

**Affiliations:** a Department of Pharmacy, Faculty of Pharmacy, Mahidol University, Bangkok, Thailand; b Department of Pharmaceutical Care, School of Pharmaceutical Sciences, University of Phayao, Phayao, Thailand; c Department of Biostatistics, Faculty of Public Health, Mahidol University, Bangkok, Thailand; d Samrejvittaya School, Sakaeo, Thailand; e College of Pharmacy, QU Health, Qatar University, Doha, Qatar

**Keywords:** Risk prediction, clinical prediction model, risk score, drug-related problem, patient safety

## Abstract

**Background:**

Drug-related problems (DRPs) are a major health concern, with half being preventable and potentially resolvable through the application of pharmaceutical care (PC). However, performing PC to all hospitalised patients is unfeasible due to staff shortages coupled with an increasing number of patients. Hence, a risk score for identifying patients at high risk of DRPs is needed. This study aimed to develop and validate a DRP risk score for hospitalised patients.

**Method:**

A prospective cohort study was conducted in a tertiary hospital in Northern Thailand. Adult patients (≥ 18 years) admitted to medical wards were included. DRPs were identified by clinical pharmacists specialising in internal medicine. Multivariable logistic regression analysis was used to construct a risk score. The score was validated using bootstrapping, and three risk groups were created based on both probability and severity. Score performance was assessed with the area under the receiver operating characteristic curve (AUROC), calibration plot, sensitivity, and specificity.

**Results:**

Among 1350 eligible admissions, 155 (11.48%) experienced at least one clinically preventable DRP. The DRP risk score included 6 predictors, namely age ≥ 65 years, chronic cardiac disease, number of drugs used prior to admission, parenteral administration (excluding parenteral nutrition), drugs with special instructions, and drugs with a high potential for drug–drug interactions. The AUROC was 0.709 (95% CI 0.672, 0.751), with good calibration (calibration slope of 0.928, intercept 0.004). Patients with a score < 4 were classified as low risk, while score ≥ 8 indicated high risk. A score of 4 yielded a sensitivity of 93.55% and a specificity of 34.48%, whereas a score of 8 demonstrated a sensitivity of 43.87% and a specificity of 83.01%.

**Conclusions:**

The DRP risk score has the potential to identify patients at risk of DRPs. External validation is needed to enhance its generalisability. Integration into automated systems may support timely pharmacist interventions.

## Background

Patient safety is a global challenge for healthcare systems and is potentially compromised by drug-related problems (DRPs) (Panagioti et al., 2024; World Health Organization, 2024a). DRPs are events involving drug therapy that actually or potentially interfere with optimal clinical outcomes (Hepler & Strand, 1990). They lead to substantial morbidity, mortality, prolong hospital stays, and increased economic burden (Hoonhout et al., 2010; Leendertse et al., 2011; Panagioti et al., 2024). Hospitalised patients are particularly vulnerable to DRPs due to frequent changes in treatment regimens, the complexity of pharmacotherapy, and dynamic clinical conditions such as organ dysfunction. These factors can alter pharmacokinetic and pharmacodynamic properties and lead to drug accumulation (Blix et al., 2004; Garin et al., 2021; Guignard et al., 2015). Previous studies have reported that the incidence of DRPs among hospitalised patients ranged from 15.5 to 81.0%, with half being preventable (Garin et al., 2021; Panagioti et al., 2019; Tharanon et al., 2022). Of note, the World Health Organization (WHO) launched the Global Patient Safety Flagship to reduce avoidable medication harm by 50% (WHO, 2024a). Additionally, improving access to safe and effective medications supports Sustainable Development Goal 3, which promotes good health and well-being for all ages (World Health Organization, 2024b). Pharmaceutical care is a key strategy to detect, resolve, and prevent DRPs (Hepler & Strand, 1990; Kaboli et al., 2006). Clinical pharmacists play a major role in optimising drug therapy and enhancing patient safety (Kaboli et al., 2006; Viktil & Blix, 2008). However, the consistent delivery of comprehensive PC to all patients is challenged by workforce shortages, increasing number of patients admissions, and shorter patient stays (Munday & Forrest, 2016; Ogbonna et al., 2015; South East England Specialist Pharmacy Services, 2018). Our tertiary care, with approximately 800 beds, serves as a referral centre for complex cases from its own province and two neighbouring provinces, resulting in a high annual admissions volume (approximately 100,000 admissions). Despite this demand, the institution faces a significant shortage of clinical pharmacists, with only 60 currently employed. This limitation affects the capacity to provide continuous PC to all patients in the medical ward. These findings highlight the need to prioritise PC by identifying patients at risk of DRPs and stratifying them based on risk level and clinical severity. This approach facilitates earlier detection and enables more timely, targeted interventions according to patient risk (Munday & Forrest, 2016; South East England Specialist Pharmacy Services, 2018).

Clinical prediction tools combine multiple important predictors (e.g. patient-related, medication-related, and laboratory-related) to estimate the risk of adverse outcomes and guide timely decision-making (Steyerberg et al., 2013). Several tools have been developed to identify hospitalised patients at risk of adverse drug reactions (ADRs), adverse drug events (ADEs), medication errors (MEs), and DRPs. These tools vary in clinical settings, methodologies, and target populations. Recent research has highlighted the development of predictive tools for ADRs in neonates and children, underscoring the importance of tailored risk assessment in these groups (Leopoldino et al., 2024; Yalçın et al., 2022, 2024). However, the present study focuses on adult patients in internal medicine wards, where clinical contexts and pharmacotherapy complexities differ from pediatric care. Examples of adult-focused tools developed primarily in European countries include the GerontoNet ADR risk score (Onder et al., 2010), and BADRI score (Tangiisuran et al., 2014), were developed to identifying older inpatients who had a high risk of ADR. The Assessment of Risk Tool (Falconer et al., 2014) and the Adverse Inpatient Medication Event (Falconer et al., 2021) were developed and validated in New Zealand, using consensus methods and statistical modelling, respectively. Tools targeting DRPs included the Drug-Associated Risk Tool (Kaufmann et al., 2018), the tool by Urbina et al. (Urbina et al., 2014), and the Medicine Optimisation Assessment Tool; MOAT (Geeson et al., 2019). While these tools have facilitated more timely and targeted pharmacist interventions, they exhibit methodological limitations (Deawjaroen et al., 2022; Jung-Poppe et al., 2022). Some tools lack clearly defined predictors (Onder et al., 2010; Tangiisuran et al., 2014; Urbina et al., 2014) and some rely on predictors unavailable at the time of use (Falconer et al., 2021; Geeson et al., 2019; Tangiisuran et al., 2014). Many tools lack extensive external validation, limiting their generalisability across clinical settings (Falconer et al., 2021; Geeson et al., 2019; Kaufmann et al., 2018; Nguyen et al., 2017). Furthermore, important predictors such as the route of administration and drugs with special instructions remain underexplored, despite their association with regimen complexity, medication errors, and adverse outcomes (Deawjaroen et al., 2022; Ferreira et al., 2015; Willson et al., 2014). Another challenge to implementation is the format of existing tools. Many rely on regression equations that require electronic systems, which may not be feasible in settings with limited technological infrastructure (Falconer et al., 2021; Geeson et al., 2019; Nguyen et al., 2017; Urbina et al., 2014). Alternative formats such as point scores, graphical charts, or mobile applications may enhance usability and facilitate integration into routine clinical workflows (Bonnett et al., 2019).

Therefore, this study aims to develop and internally validate a risk score for identifying DRPs among hospitalised adult patients, using predictors that are routinely available in clinical practice. The model incorporates key predictors, including the route of drug administration and medications with special instructions. A combined approach was employed for predictor selection, integrating expert clinical judgment with statistical modelling techniques to enhance the clinical relevance of the tool.

## Methods

### Study design and participants

This was a prospective cohort study conducted in an 800-bed tertiary hospital in Northern Thailand. Eligible patients were consecutively enrolled between January and July 2020. Adult patients aged ≥ 18 years admitted to general medical wards and hospitalised for more than 24 hours were included. Patients were excluded if their prescribing records were not reviewed by a clinical pharmacist during admission, as DRP identification could not be confirmed.

### Sample size

The sample size was estimated using the event per variable (EPV) ratio of 10 (Moons et al., 2014; Peduzzi et al., 1996). At least one hundred-fifty DRP were needed to evaluate 16 predictors in multivariable analysis.

### Data collection

The outcome was the occurrence of at least one clinically preventable DRP. DRPs were identified by the principal investigator and clinical pharmacists as part of their routine daily assessments and evaluated continuously throughout the study period. Assessors were pharmacists specialised in internal medical and had a minimum of three years of clinical experience. Each identified DRP was subsequently evaluated for causality, preventability, severity and prospectively documented throughout the hospital stay.

DRPs were defined according to the definition of Hepler and Strand (1990) as ‘an undesirable patient experience that involves drug therapy and that actually or potentially interferes with a desired patient outcome’. They were classified the problems and causes using the Pharmaceutical Care Network Europe (PCNE) Foundation classification system (Pharmaceutical Care Network Europe, 2018.). Preventability was assessed using the modified Hepler and Strand criteria, the following three elements: (1) the DRP must be recognisable, and the likelihood of an undesirable clinical outcome must be foreseeable; (2) the causes of that outcome must be identifiable and (3) those causes must be controllable. Severity was evaluated using the National Coordinating Council for Medication Error Reporting (NCC MERP) categorises events as categories A to I (National Coordinating Council for Medication Error Reporting and Prevention, 2019). DRPs rated as category C or higher were defined as clinically DRPs, indicating event that reached the patient. A senior clinical pharmacist independently reviewed and validated all DRPs assessments. Any discrepancies were resolved by consensus (NS and NP; senior clinical pharmacists).

Candidate predictors were predefined using two approaches, including a systematic review (Deawjaroen et al., 2022) and expert consensus (Supplemental Material 1), with consideration for practical applicability. Data were extracted from the hospital’s electronic database and medical chart by a principal investigator (Supplemental Material 2). Sixteen preselected predictors were: age, weight, history of drug allergy, number of comorbidities, chronic cardiac disease, hypertension, dyslipidemia, number of drugs used prior to admission, parenteral administration, drugs with special instructions, number of regular drugs prescribed, antithrombotic drug, cardiovascular drugs, antimicrobial drugs, drugs with a high potential for drug–drug interactions, and serum creatinine (Supplemental Material 3). Continuous predictors (e.g. age, number of drugs used prior to admission, and number of regular drugs prescribed) were dichotomised using clinically meaningful risk thresholds (Falconer et al., 2014; Kaufmann et al., 2018).

### Statistical analysis

Analyses were performed using Stata V.18.0 (StataCorp, College Station, Texas, USA). Descriptive statistics were reported as Frequency (percentage) for categorical variables and median with interquartile range (IQR) or mean with standard deviation (SD) for continuous variables. Missing data were identified and reported for each predictor. Predictors were excluded if missing data exceeded 20%, prevalence among cases was less than 5% or collinearity was present (correlation coefficient ≥ 0.70) (Geeson et al., 2019; Royston et al., 2009; Steyerberg et al., 2013). Complete case analysis was performed.

Univariable analysis was conducted to identify predictors of DRP, with predictors showing *p* < 0.25 included in a multivariable logistic regression (Geeson et al., 2019; Moons et al., 2014; Tangiisuran et al., 2014). The multivariable logistic regression was performed using the backward elimination process to retain independent predictors with a *p*-value < 0.157 (Moons et al., 2014; Steyerberg, 2009). Results are reported as odds ratio (OR) with 95% confidence intervals (95% CI). Internal validation was performed using bootstrapping (500 replications) and a shrinkage factor was applied to adjust regression coefficients for model optimisation (Moons et al., 2014; Steyerberg, 2009). Model performance was evaluated using calibration and discrimination. A well-calibrated model aligns with the 45-degree line, with an intercept of 0 and slope of 1. Discrimination was evaluated using the AUROC, interpreted as excellent (0.90–1.00), good (0.80–0.89), fair (0.70–0.79), poor (0.60–0.69), or failed (<0.60) (Steyerberg, 2009). A simple score system was constructed by rounding adjusted OR. Predictors with aOR of 1.00–1.99 received 1 point, 2.00–2.99 received 2 points, and so on (Urbina et al., 2014). The total score was computed by summing individual predictor scores. Patients experiencing DRPs were categorised into three risk groups (low, moderate, and high risk) based on both probability and severity. Cut-off thresholds were informed by a pharmacist survey and adapted from Geeson et al (Geeson et al., 2019). Cut-off scores were selected to achieve 80% sensitivity between low and moderate risk groups, and 50% sensitivity between moderate and high risk groups.

This study was approved by the Research Ethics Committee of Institutional Review Board (MU-DT/PY-IRB 2019/067.0110) and the Research Ethics Board of hospital (No.107/62). The requirement for patient consent was waived, as the study utilised secondary data from medical records and hospital databases collected during routine care. All processes adhered to the Declaration of Helsinki and institutional ethical standards. The study was reported in accordance with the TRIPOD guidelines (Collins et al., 2015).

## Results

### Study participants

During the study period, 1510 patient admissions were admitted to general medical wards, and 95 of these admissions were excluded due to admission of less than 24 hours. According to the definition of study outcome, a total of 1350 patient admissions with 155 (11.48%) clinically preventable DRPs were included in the model development (Supplemental Material 4). The characteristics of the 1350 patient admissions are summarised in Table 1. The mean age was 64.3 ± 16.1 years and 62.5% were male. Most patients (81.7%) had comorbidities, with approximately half taking at least 5 drugs per day.

**Table 1.** Characteristics of study admissions

Characteristic	Admissions without DRP, (*n* = 1195)	admissions with DRP (*n* = 155)	All admissions (*n* = 1350)
**Patient-related**			
Male, *n* (%)	742 (62.1)	102 (65.8)	844 (62.5)
Age, mean (SD)	63.7 (16.0)	69.0 (15.8)	64.30 (16.1)
Weight (kg), mean (SD)	55.4 (13.2)	53.3 (13.0)	55.1 (13.2)
History of readmissions*, *n* (%)	439 (36.7)	81 (52.3)	520 (38.5)
Number of previous admissions in previous 6 months*, median (IQR)	1 (2) Range: 1-17	1 (1) Rang: 1-8	1 (2) Range: 1-17
Number of comorbidities, mean (SD)	2.0 (1.6)	2.8 (1.5)	2.1 (1.6)
Comorbidities, *n* (%)	956 (80.0)	147 (94.8)	1103 (81.7)
Chronic cardiac disease	171 (14.3)	47 (30.3)	218 (16.2)
Renal disease	211 (17.7)	40 (25.8)	251 (18.6)
Chronic liver disease	59 (4.9)	5 (3.2)	64 (4.7)
Diabetes	287 (24.0)	42 (27.1)	329 (24.4)
Hypertension	571 (47.8)	100 (64.5)	671 (49.7)
Dyslipidaemia	391 (32.7)	68 (43.9)	459 (34.0)
**Type of admission, *n* (%)**			
Emergency	712 (59.58)	106 (68.39)	818 (60.59)
Schedule	107 (8.95)	17 (10.97)	124 (9.19)
Referral	376 (31.46)	32 (20.65)	408 (30.22)
Previous drug allergies, *n* (%)	123 (10.3)	22 (14.2)	146 (10.8)
Length of stay in days; median (IQR)	3 (4) Range: 1-44	4 (6) Range: 1-33	4 (4) Range: 1-44
**Drug-related**			
Drugs used prior to admission**,** *n* (%)	846 (70.8)	138 (89.9)	984 (72.9)
Number of drugs used prior to admission, mean (SD)	4.2 (3.8)	5.9 (3.7)	4.4 (3.8)
Number of drugs used prior to admission ≥ 5 items, mean (SD)	528 (44.2)	105 (67.7)	633 (46.9)
Parenteral administration**,** *n* (%)	844 (70.6)	128 (82.6)	972 (72.0)
Drugs with special instructions**,** *n* (%)	275 (23.0)	71 (45.8)	346 (25.6)
Number of regular drugs prescribed on admission, mean (SD)	4.3 (3.2)	5.6 (3.5)	4.5 (3.3)
Drugs with a high risk of adverse drug reaction, *n* (%)	849 (71.0)	127 (81.9)	976 (72.3)
Antithrombotic drugs	195 (16.3)	40 (25.8)	235 (17.4)
Cardiovascular drugs	603 (50.5)	99 (63.9)	702 (52.0)
Antibiotic drugs	50 (4.2)	10 (6.4)	60 (4.4)
Antihyperglycemic drugs	169 (14.1)	23 (14.8)	192 (14.2)
Antiepileptic drugs	36 (3.0)	7 (4.5)	43 (3.2)
High concentration electrolyte injection	258 (21.6)	35 (22.6)	293 (21.7)
Drugs with a high potential for drug-drug interactions**,** *n* (%)	197 (16.5)	54 (34.8)	251 (18.6)
Warfarin	56 (4.7)	17 (11.0)	73 (5.4)
Anticonvulsants	35 (2.9)	6 (3.9)	41 (3.0)
Antiretroviral drugs	25 (20.9)	11 (7.1)	36 (2.7)
Antifungal drugs	17 (1.4)	9 (5.8)	26 (1.9)
Antituberculous drugs	37 (3.1)	8 (5.2)	45 (3.3)
**Laboratory results**			
Serum creatinine, median (IQR)	1.0 (0.8) Range: 0.2-24.9	1.2 (1.2) Range: 0.2-16.0	1.0 (0.9) Range: 0.2-24.9
Liver abnormal, *n* (%)	150 (16.0)	19 (15.1)	169 (15.9)
Serum albumin, mean (SD)	3.2 (0.8)	3.2 (0.7)	3.2 (0.7)
Serum sodium, mean (SD)	136.7 (6.0)	136.3 (6.4)	136.6 (6.1)
Serum potassium, mean (SD)	3.9 (0.8)	3.9 (0.7)	3.9 (0.7)
Haemoglobin, mean (SD)	11.0 (2.8)	10.6 (2.5)	10.9 (2.8)
White cell count, mean (SD)	9.9 (6.1)	10.4 (5.9)	10.0 (6.1)
INR, median (IQR)	1.1 (0.3) Range: 0.8-7.3	1.1 (0.3) Range: 0.8-10.6	1.1 (0.3) Range: 0.8-10.6

Notes: DRP drug-related problem, IQR = interquartile range, NSAIDs = non-steroidal anti-inflammatory drugs, SD = standard deviation,

### Characteristics of identified DRP

A total of 316 preventable DRPs occurred in 271 admissions, with a median of 1 (range 1–3) DRP per patient. There was no disagreement between independent investigators regarding the assessment of DRP. According to the PCNE V8.02 classification, the principal categories of DRPs were treatment safety (45.8%, *n* = 71), followed by treatment effectiveness (38.1%, *n* = 59). Within treatment effectiveness, untreated symptoms or indications (22.6%, *n* = 35) were the major DRPs. Regarding the causes of the DRPs, the predominant category identified was dosage selection, with a dose too high (32.9%, *n* = 51). Drug selection was the next most frequently reported, including no drug treatment despite existing indication and inappropriate duplication of therapeutic group, accounting for 24.5% and 7.7% of the total of DRPs, respectively.

### Model development

Fourteen predictors were a statistically significant associated with DRP (*p*-value < 0.25) (Table 2). Although the history of ADR was not statistically significant, it was retained as a predictor and included in the multivariable logistic regression analysis due to its clinical relevance and presence in previously published risk models (Falconer et al., 2021; Geeson et al., 2019). After performing multivariable logistic regression analysis, 6 predictors remained associated with the risk of DRP (*p*-value < 0.157). The point assigned to each predictor in the DRP risk score is shown in Table 2. The score ranged from 0 to 12.

**Table 2.** Predictors associated with the occurrence of DRP in hospitalised patients.

Predictor	Univariable analysis			Multivariable analysis Adjusted final Model*			Point
	OR	95% Cl	*P* value	aOR	95% Cl	*P* value	
Age ≥ 65 years	2.05	1.41, 2.97	< 0.001	1.71	1.20, 2.42	0.003	2
Weight	0.98	0.97, 0.10	0.044	–	–	–	
History of ADR	1.31	0.77, 2.22	0.326	–	–	–	
Number of comorbidities	1.35	1.22, 1.51	< 0.001	–	–	–	
Chronic cardiac disease	1.56	1.02, 2.38	0.040	1.57	1.06, 2.32	0.024	2
Hypertension	2.03	1.41, 2.94	< 0.001	–	–	–	
Dyslipidaemia	1.72	1.20, 2.45	0.003	–	–	–	
Number of drugs used prior to admission ≥ 5 items	2.59	1.78, 3.76	< 0.001	1.60	1.10, 2.31	0.013	2
Parenteral administration	1.97	1.28, 3.04	0.002	1.88	1.24, 2.84	0.003	2
Drugs with special instructions	2.83	2.01, 3.99	<0.001	1.76	1.22, 2.52	0.002	2
Number of regular drugs prescribed ≥ 5 items	2.19	1.49, .22	< 0.001	–	–	–	
Antithrombotic drugs	1.57	1.02, 2.24	0.012	–	–	–	
Cardiovascular drugs	1.75	1.23, 2.50	0.002	–	–	–	
Antimicrobial drugs	1.48	1.04, 2.11	0.029	–	–	–	
Drugs with a high potential for drug-drug interactions ^#^	2.43	1.66, 3.55	< 0.001	1.86	1.28, 2.72	0.001	2
Serum creatinine (log form)	1.24	1.00, 1.54	0.051	–	–	–	
Constant				0.02	0.02, 0.22		

Notes: aOR = adjusted odds ratio, ADR = adverse drug reaction, DI = drug interaction, 95% CI = 95% confidence interval.

# Drugs with a high potential for drug-drug interactions including warfarin, anticonvulsants, antiretroviral drugs, antifungal drugs, antituberculous drugs.

* Model adjusted by bootstrap shrinkage factor (0.928).

– not included in the final model.

### Model performance

**Figure 1.**
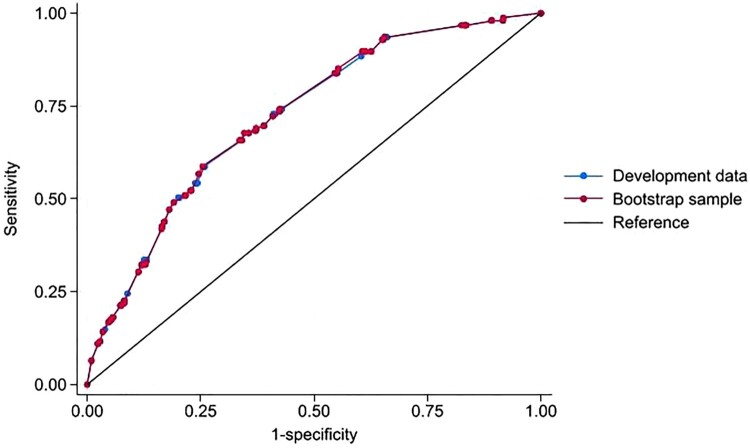
AUROC for the DRP risk score between the development sample and bootstrap validation sample.

**Figure 2.**
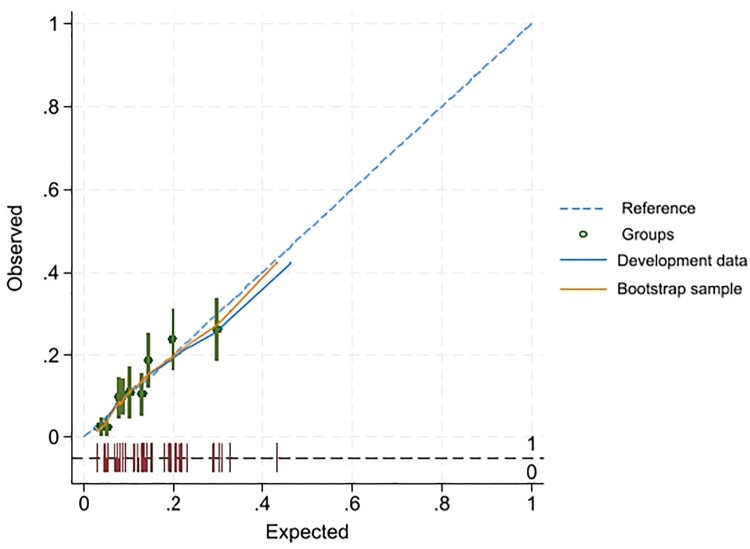
Calibration plot for the DRP risk score between the development sample and bootstrap validation sample.

The AUROC for the development data was 0.721 (95% CI 0.682, 0.762), with good calibration (calibration slope 0.991, intercept 0.008). When applied to the bootstrap sample, model performance slightly reduced to 0.709 (95% CI 0.672, 0.751), with constantly good calibration (calibration slope 0.928, intercept 0.004) (Figures 1 and 2).

The sensitivity of the DRP risk score using the decision threshold (score of 4) separating low-risk and moderate-risk patients was 93.55% and the specificity was 34.48%. At the threshold (score of 8) categorising patients between moderate-risk and high-risk, the sensitivity was 43.87% and the specificity was 83.01%.

## Discussion

The DRP risk score can estimate the occurrence of DRP in hospitalised patients based on their risk, which has the potential to be applied to patients across a wide range of patients in terms of age, and medical conditions. It will assist hospital pharmacists in conducting interventions in a more timely and targeted manner during situations of staff shortage. Our score is based on simple predictors available in the medical records and can be easily calculated. It consists of 6 predictors, namely age ≥ 65 years, chronic cardiac disease, number of drugs used prior to admission, parenteral administration, drugs with special instructions, and drugs with a high potential for drug–drug interactions. The performance of the score demonstrated fair discrimination and good calibration.

There are 6 predictors included in the DRP risk score. This set of patient information reflects the complexity of patient care, aiding in predicting DRP risk. Most of these predictors in the DRP risk score have previously been associated with DRP (Falconer et al., 2014; Geeson et al., 2019; Meid et al., 2018; Urbina et al., 2014). Compared to previous studies, the different predictors retained in the final model may also represent the uniqueness of the patient population studied and the pattern of drug use. Most common drug-related predictors, such as antithrombotic and cardiovascular drugs, and history of drug allergy, were not included in the new DRP risk score because they did not significantly contribute to DRPs in our study population, despite being identified as significant risk factors in previous studies (Falconer et al., 2021; Geeson et al., 2019). This is the first time that parenteral administration and drugs with special instructions have been identified as significant predictors in the DRP risk score. It can be explained by dose adjustments, drug incompatibilities, and adverse drug effects profiles, especially for drugs given via intravenous route. Drugs with special instructions, such as specific timing of administration can result in medication regimen complexity. Complex regimens are challenging for both patients and staff to manage, and may increase the risk of nonadherence and drug administration errors, such as administering medications at the wrong time (Chen et al., 2019; Ferreira et al., 2015; Willson et al., 2014).

Overall DRP risk score performance was acceptable both in terms of discrimination (AUROC > 0.6) and calibration (slope of calibration plot of closely to one and intercept closely to 0) (Steyerberg, 2009). This performance was consistent with existing tools, such as MOAT (AUROC 0.681, 95% CI: 0.654 to 0.708) (Geeson et al., 2019), Urbina et al. (2014) (AUROC 0.776, 95%CI: 0.759, 0.792), and Nguyen et al. (2017) (AUROC 0.718, 95% CI: 0.689–0.748). The score displayed good calibration, with calibration intercept ranging from −0.005 to 0.010, and slop ranging from 0.891 to 1.004, which comparable to the MOAT (Geeson et al., 2019). While machine learning (ML) techniques have demonstrated higher AUROC values than logistic regression tools (Langenberger, 2023; Yalçın et al., 2023), their implementation often requires complex infrastructure. Our study aimed to develop a simple bedside risk score for clinical pharmacists, without requiring computational infrastructure. Conducted in a single tertiary hospital with a limited sample size, logistic regression was more suitable given contextual constraints. ML models were inherently data-driven and developed using large-scale data, resulting in naturally enhanced predictive performance. However, it may overfit when applied to smaller or less diverse datasets (Langenberger, 2023; van der Ploeg et al., 2014; Yalçın et al., 2023). Given these considerations, logistic regression was selected as the modelling approach in our study. Future research should investigate hybrid approaches that balance predictive performance with clinical usability and relevance.

To be a user-friendly and clinically applicable score, the major predictors included in the DRP risk scores were routinely recorded and available at the time of use. This helped to easily incorporate into clinical practice without added burdens. The score provides simple calculations for converting clinical risk factors into scores. Total score is categorised into three risk groups including low, moderate, and high risk of DRP, guiding general prioritisation decisions. It can be used easily and quickly to interpret patient risk in clinical settings, facilitating its use at the bedside and enabling the efficient, patient-specific allocation of clinical pharmacy services (Bonnett et al., 2019; Geeson et al., 2019; Steyerberg, 2009).

Accuracy of the score showed a high sensitivity of 93.55% to distinguish patients at low risk and moderate risk of DRP (a cut-off score of 4), a level of accuracy deemed acceptable by a previous study (Geeson et al., 2019). The sensitivity of approximately 90% indicates that the score is able to identify 90% of patients likely to experience moderate or severe DRP. While its specificity of 34.48% at low and moderate risk threshold was low. It means that our score may fail to identify patients at risk of a moderate or severe DRP, while incorrectly categorising low risk of DRP as moderate or severe DRP. However, a false positive classification (1-specificity) would not seriously affect the patient’s outcome as the main aim of the score is to identify patients at high risk of DRP for given timely pharmacist intervention. At a cut-off score of 8, the model showed a sensitivity of 43.87% and specificity of 83.01% for identifying patients at moderate to severe DRP risk. However, the values of sensitivity and specificity can vary depending on the chosen threshold, which should be tailored to the intended use, organisational need, and resources of staff providing interventions.

Our study was conducted according to PROGRESS (Steyerberg et al., 2013) and reported following TRIPOD (Collins et al., 2015) statements. Both are widely recognised and recommended for the development and reporting of clinical prediction models. Importantly, we acknowledge the limitations of relying solely on univariable associations for predictor selection. As highlighted in the literature, this approach can introduce selection bias and increase the risk of overfitting, potentially leading to the exclusion of predictors that may only demonstrate significance after adjustment for other covariates. To overcome this, we employed a structured and transparent approach to predictor selection, incorporating three strategies: (1) univariable analysis; (2) previously published significant risk factor; and (3) clinical considerations. This combined strategy ensures that the selected predictors are both statistically and clinically relevant, while also minimising bias and enhancing the robustness of the model.

The predictors in the DRP risk score are routinely collected from inpatient data and can be available at the time of its intended use. This characteristic enhances the applicability of the score to practice, to avoid the need for additional measurements and avoided the need for complex calculations (Abuzour et al., 2021; Lee et al., 2016). Furthermore, we performed bootstrapping along with a shrinkage factor to address model overfitting (adjusting for overoptimistic predictions). Bootstrapping is more efficient and generally recommended for estimating the internal validity of a logistic regression model, especially when the sample size is small and external validation is limited (Steyerberg et al., 2001).

There are some limitations of this work. First, the prospective study was conducted in a tertiary hospital. Some patients were referred from primary care or other hospitals, resulting in missing values of predictors (such as history of previous admission or some baseline of laboratory). Thus, these predictors were excluded from the set of candidate predictors. Second, this study was conducted in one sitting. Included predictors may be represented based on the characteristics of the study population, which might be different from other settings and limit its generalisability to other institutions. Further studies are needed to externally validate this risk score in different populations and settings prior to implementation.

## Conclusion

The study developed a clinically relevant DRP risk score using key patient and medication factors, particularly drugs with special instructions and parenteral administration, which are significantly associated with DRPs during hospitalisation. The score demonstrated good discrimination and calibration, supporting targeted pharmacist intervention. External validation and integration into electronic health record systems are needed to enable timely risk identification and early pharmacist intervention.

## Supplementary Material

Supplemental Material 1

Supplemental Material 3

Supplemental Material 4

Supplemental Material 2
