# Supplementary material for: Development and validation of a clinical score to identify hospitalised patients at high risk of drug-related problems
Source: J Pharm Policy Pract. 2025 Sep 23;18(1):2557876. doi: 10.1080/20523211.2025.2557876 (PMC12459166; doi:10.1080/20523211.2025.2557876)
Supplement: Supplemental Material 1 [file JPPP_A_2557876_SM1116.docx]

**Supplement 1 Result of hospital pharmacist survey**

The questionnaires were distributed to 28 hospitals across Thailand, consisting of hospitals under the Ministry of Public health and Ministry of Education. The factors set in the median range at 4 or more of the score given by hospital pharmacists’ opinion and the list of drugs selected by hospital pharmacists more than 50%, were included as candidate predictors

| Predictor | Literature reviews (previous clinical prediction tools) | Survey results | | Available when model intended to be used | Clearly defined | Included in standard clinical datasets  (Expected data available > 50%) | **Selected as a candidate predictor** |
| --- | --- | --- | --- | --- | --- | --- | --- |
|  |  | Median response* | Interquartile range |  |  |  |  |
| **Patient related** |  |  |  |  |  |  |  |
| Age | M | 5 | 1 | **/** | **/** | **/** | **/** |
| Gender | M | 3 | 1 | **/** | **/** | **/** | **/** |
| History of drug allergy | M | 5 | 0 | **/** | **/** | **/** | **/** |
| History of admissions within 30 days | U, ++ | 5 | 1 | **/** | **/** | **/** | **/** |
| Number of admissions in previous 6 months | U, ++ | 4 | 1 | **/** | **/** | **/** | **/** |
| Number of comorbidities | M | 5 | 1 | **/** | **/** | **/** | **/** |
| Type of Comorbidities | M | 5 | 1 | **/** | **/** | **/** | **/** |
| chronic cardiac disease | M, ++ | - | - | **/** | **/** | **/** | **/** |
| renal disease | M, ++ | - | - | **/** | **/** | **/** | **/** |
| chronic liver disease | M, ++ | - | - | **/** | **/** | **/** | **/** |
| chronic respiratory disease | ++ | - | - | **/** | **/** | **/** | **/** |
| diabetes | ++ | - | - | **/** | **/** | **/** | **/** |
| Hypertension | U | - | - | **/** | **/** | **/** | **/** |
| Dyslipidemia | M | - | - | **/** | **/** | **/** | **/** |
| Pathway of admission such as Emergency, schedule, referral | M, ++ | 4 | 2 | **/** | **/** | **/** | **/** |
| Diagnosis/ reason for admission | M | 4 | 1 | X | / | / | X |
| Transition of care | ++ | 4 | 1 | X | / | / | X |
| **Medication related** |  |  |  |  |  |  |  |
| Number of drugs prescribed | M, ++ | 5 | 1 | **/** | **/** | **/** | **/** |
| Type of drug prescribed | M | 5 | 1 | **/** | **/** | **/** | **/** |
| Use of high-risk medicines causing adverse reactions: | M | 5 | 1 | **/** | **/** | **/** | **/** |
| Antithrombotic agents | M, ++ | > 50% | > 50% | **/** | **/** | **/** | **/** |
| Cardiovascular drugs | M, ++ | > 50% | > 50% | **/** | **/** | **/** | **/** |
| Antimicrobial drugs | M, ++ | > 50% | > 50% | **/** | **/** | **/** | **/** |
| Antihyperglycemic drugs | M, ++ | > 50% | > 50% | **/** | **/** | **/** | **/** |
| Antiepileptic drugs | M, ++ | > 50% | > 50% | **/** | **/** | **/** | **/** |
| High Concentration electrolyte |  | > 50% | > 50% | **/** | **/** | **/** | **/** |
| Use of high-risk medicines causing serious drug-drug interactions | M | 5 | 1 | **/** | **/** | **/** | **/** |
| Warfarin | M, ++ | > 50% | > 50% | **/** | **/** | **/** | **/** |
| Anticonvulsants | M, ++ | > 50% | > 50% | **/** | **/** | **/** | **/** |
| Antiretroviral drugs | M, ++ | > 50% | > 50% | **/** | **/** | **/** | **/** |
| Antitubercular drugs | M, ++ | > 50% | > 50% | **/** | **/** | **/** | **/** |
| Parenteral administration | ++ | 4 | 1 | **/** | **/** | **/** | **/** |
| Special instructions | ++ | 5 | 1 | **/** | **/** | **/** | **/** |
| **Laboratory results** |  |  |  |  |  |  |  |
| Renal function | M | 5 | 0 | **/** | **/** | **/** | **/** |
| Liver disease | U | 5 | 1 | **/** | **/** | **/** | **/** |

++ refer to important based on consensus tool studies or previous survey

* Likert responses 5 = very important, 4 = important

M = factor was significant in multivariate analysis, U = factor was significant in univariate analysis

> 50% = A drug was selected by > 50% of respondents

X = factor was not considered to be preselected predictor due to such reasons

/ = factor was preselected predictor due to such reasons
