# Supplementary material for: Development and validation of a clinical score to identify hospitalised patients at high risk of drug-related problems
Source: J Pharm Policy Pract. 2025 Sep 23;18(1):2557876. doi: 10.1080/20523211.2025.2557876 (PMC12459166; doi:10.1080/20523211.2025.2557876)
Supplement: Supplemental Material 3 [file JPPP_A_2557876_SM1115.docx]

**Supplement 3** Preselected predictors for the development of DRP risk score

| **Factors** | **Type of measurement** | **Number of variables** |
| --- | --- | --- |
| Age ≥ 65 years | Binary | 1 |
| Weight | Continuous numeric | 1 |
| History of drug allergy | Binary | 1 |
| Number of comorbidities | Continuous numeric | 1 |
| Type of comorbidity  - Chronic cardiac disease,  - Hypertension,  - Dyslipidaemia | Binary | 3 |
| Number of drugs used prior to admission ≥ 5 items | Binary | 1 |
| Parenteral administration | Binary | 1 |
| Drugs with special instructions | Binary | 1 |
| Number of regular drugs prescribed ≥ 5 items | Binary | 1 |
| Drugs with a high-risk of causing ADR  - Antithrombotic drugs  - Cardiovascular drugs  - Antimicrobial drugs | Binary | 3 |
| Drugs with a high potential for drug-drug interactions including warfarin, anticonvulsants, antiretroviral drugs, antifungal drugs, antituberculous drugs | Binary | 1 |
| Serum creatinine | Continuous numeric | 1 |
