# Supplementary material for: Development and validation of a clinical score to identify hospitalised patients at high risk of drug-related problems
Source: J Pharm Policy Pract. 2025 Sep 23;18(1):2557876. doi: 10.1080/20523211.2025.2557876 (PMC12459166; doi:10.1080/20523211.2025.2557876)
Supplement: Supplemental Material 4 [file JPPP_A_2557876_SM1114.doc]

**Supplement 4** Flowchart of study

NCC MERP Cat A = 0

NCC MERP Cat B = 116

NCC MERP Cat C = 116

NCC MERP Cat D = 39

NCC MERP above Cat D = 0

Number of patient admissions

Occurring DRP, n = 300

Patient admissions experiencing DRP

in hospital, n = 271

Excluded n = 36

No available history of medication use (20),

no data of wight/Hight (16)

Excluded n = 29

DRP related admission (26), not preventable DRP (3)

Total number of patient admissions screened over 7 months, n = 1510 admission

Eligible patient admissions

n = 1415

Number of patient admissions

no DRP, n = 1115

Excluded n = 95

admission ≤ 24 hr.

Non DRP included in analysis

n = 1195

Admission without DRP included in the final analysis, n = 1079

Admissions with occurring DRP category C and above included in analysis, n = 155

Admissions with occurring DRP category B, n = 116
