# Supplementary material for: Development and validation of a clinical score to identify hospitalised patients at high risk of drug-related problems
Source: J Pharm Policy Pract. 2025 Sep 23;18(1):2557876. doi: 10.1080/20523211.2025.2557876 (PMC12459166; doi:10.1080/20523211.2025.2557876)
Supplement: Supplemental Material 2 [file JPPP_A_2557876_SM1113.docx]

**Supplement 2** Candidate variable definitions

| **Candidate predictors** | **Details** |
| --- | --- |
| Age | Age at admission to hospital (years) |
| Gender | Male and female |
| Weight | Weight at first documented data following admission. If no data were available, data was extracted from previous admission. Weight data were accepted within one month prior. |
| History of drug allergy | Obtained from medical record/ electronic databases and recorded whether patients had history of ADR or drug allergy |
| Pathway of admission | It is composed of 3 categories, including emergency, schedule, referral |
| Transition of care | Patient was transferred from other ward to general medical wards |
| History of hospital admission, number of hospital admissions | History and number of hospital admission in 6 months prior to admission, including emergency department, any type of departments |
| Presence of comorbidity, Number of comorbidities | Data were obtained from hospital clinical coding data (ICD-10 codes) in electronic databases or medical record charts |
| Type of comorbidity | Data were obtained from hospital clinical coding data (ICD-10 codes) in electronic databases or medical record charts. Grouped into:  1. Chronic cardiac disease (heart failure, coronary heart disease, arrhythmias)  2. Renal impairment (chronic dialysis or renal transplantation, eGFR < 30 ml/min/1.73m^2^)  3. Chronic liver disease (diagnosis of hepatic impairment and/or chronic hepatitis and/or hepatic cirrhosis)  4. Chronic respiratory disease (diagnosis of asthma, chronic obstructive pulmonary disease)  5. Diabetes type 1 or 2  6. Dementia or central nervous system impairment |
| Number of drugs used prior to admission | Number of regular drugs used prior to admission |
| Number of regular drugs prescribed | Number of regular drugs prescribed to be given during hospital stay, excluding “when required”, “once only or stat dose” drugs, topical drugs, dietary products. The count included criteria as follows:  1. combination drugs were counted as one drug  2. the count included all drugs prescribed as once weekly medication or twice weekly, except drug withheld for clinical reasons.  3. more than one formulation of the same drugs (e.g., standard and modified release drugs) were prescribed in same route, they were counted as one drug. |

**Supplement 2** Candidate variable definitions (cont.)

| **Candidate predictors** | **Details** |
| --- | --- |
| Parenteral administration route | Administration of regular drugs via intravenous, intramuscular, subcutaneous, excluding fluid replacement and nutrition parenteral. At least one drug given via any route was recorded as patients used parenteral administration route |
| Drugs with special instructions | Use of drugs with special instructions such as special times (e.g., taking doses every other day, take doses exactly 12 hours apart: 2 tablets at 08.00 a.m. and 08.00 p.m., taking doses at least 1 hour before meals, take with food) |
| Drugs with a high potential for drug-drug interactions | Drugs were prescribed as a regular drug during hospital stay, including   1. Warfarin 2. Anticonvulsants: phenytoin, carbamazepine,   phenobarbital   1. Antiretroviral drugs 2. Antifungal drugs: ketoconazole, itraconazole, fluconazole, voriconazole   5. Antitubercular drugs |
| Drugs with a high-risk of causing ADR | Drugs were prescribed as a regular drug during hospital stay, including  1. Antithrombotic drugs: anticoagulant drugs and thrombolytic drugs  2. Cardiovascular drugs: digoxin, amiodarone, antihypertension drug (calcium channel blockers, ACEIs/ARBs, beta/alpha blockers), Antiplatelet agents  3. Antimicrobial drugs: amikacin, gentamicin, amphotericin B, colistin, fosfomycin, vancomycin  4. Antihyperglycemic drugs: Insulin and sulfonylureas  5. Antiepileptic drugs:  6. High concentration electrolyte: MgSO4 injection, KCI injection, Calcium injection |
| Renal function | Serum creatinine (mg/dl) reported for the first-time following admission |
| Abnormal liver function | Abnormal liver function was defined as ALT/AST and/or bilirubin ≥ 3-time normal range. The results reported for the first following admission were considered. If no data were available, documented liver disease from medical record chart was used to define. |
| Other laboratory results | Serum albumin (g/dl), serum sodium (mEq/L), serum potassium (mEq/L)  hemoglobin (g/dl), white blood cell counts (cell/m.m^3^), platelet counts (/m.m^3^)  International normalized ratio (INR) |

ACEIs = angiotensin-converting enzyme inhibitors, ADR = adverse drug reaction, ALT = alanine aminotransferase, AST **=** aspartate aminotransferase, ARBs **=** angiotensin receptor blockers
